# Risk of child undernutrition in households with life-limiting illness: A cross-sectional study

**DOI:** 10.4102/phcfm.v17i1.4773

**Published:** 2025-03-12

**Authors:** Janni B. Petersen, Irene Naleba, Josephine Namugambe, Sofine Heilskov, Per Kallestrup

**Affiliations:** 1 Department of Public Health, Faculty of Health, Aarhus University, Aarhus, Denmark; 2 Emergency Department, Regional Hospital Horsens, Horsons, Denmark; 3 Rays of Hope Hospice Jinja, Jinja, Uganda; 4 Department of Clinical Medicine, Faculty of Health, Aarhus University, Aarhus, Denmark

**Keywords:** acute malnutrition, stunting, palliative care, household dietary diversity, household food insecurity

## Abstract

**Background:**

An ongoing challenge within the field of undernutrition is to identify children at risk.

**Aim:**

The objective of this study was to investigate whether children who are living in households inhabiting a household member with a life-limiting illness are at risk of undernutrition.

**Setting:**

A comparative cross-sectional study was performed in Uganda.

**Methods:**

We collected anthropometric data on children under the age of five and information on household dietary diversity, food security and healthcare barriers. Study participants for the hypothesised high-risk group were recruited within households receiving home-based palliative care. The comparison group included neighbouring households.

**Results:**

Data collection from 145 paired households was performed from April to July 2021. There was no statistically significant difference in prevalence of undernutrition. For all continuous nutrition indicators there was a trend towards less undernutrition in the hypothesised high-risk group than in the comparison group. We found lower overall prevalence of acute malnutrition than expected. The hypothesised high-risk group was associated with higher food insecurity than the comparison group.

**Conclusion:**

Lower overall acute malnutrition than expected may be because of the season variability. Stunting was higher than expected in both groups, which may suggest season variability in undernutrition. The results suggest a protective effect of receiving home-based palliative care on child nutritional status.

**Contribution:**

The study did not show a risk of child undernutrition in households inhabiting a household member with life-limiting illness. Future research may identify key elements responsible for the potential protective effects of home-based palliative care on child undernutrition.

## Introduction

The global problem of malnutrition is addressed in the United Nations’ sustainable development goal 2, which aims to end hunger, achieve food security and improved nutrition and promote sustainable agriculture. The goal further places special emphasis on the poor and people in vulnerable situations, including infants. In addition, target goal 2.2 describes the aim to end all forms of malnutrition by 2030, including achieving, by 2025, the internationally agreed targets on stunting and wasting in children under 5 years of age.^1,2^

Reaching these goals demands tremendous efforts. One challenge is to establish data, which provides the foundation for relevant interventions. Both the Global Nutrition Report 2018 and Uganda’s Country Strategic Plan (CSP) (2018–2022) for the World Food Programme underline the need for disaggregated data on a subnational level.^3^

One of the strategic outcomes in the CSP is ‘Refugees and other crisis-affected people have access to adequate nutritious food in times of crisis’.^3^ One risk group that possibly falls under this category is households containing members with severe, life-limiting illness and need of palliative care.

When a household member falls severely ill to such a degree that palliative care is needed, the entire household is affected by their illness. Besides the psychological and emotional stress, the household is also affected in terms of a restrained economy because of higher healthcare expenses and an increased demand for caretakers of the household. The higher demands to healthcare expenses and caretaking in a household increase the risk of poverty and food insecurity, which in turn has been demonstrated to pose a risk of child undernutrition.^4^ However, another study has shown that household level factors do not have a statistically significant impact of relapse of child acute malnutrition,^5^ which indicates that the influence of altered household circumstances poses only a small risk of child undernutrition. The diverging evidence calls for further research in household factors on the risk of child undernutrition.

Currently community-based guidelines are provided by World Health Organization (WHO)^6^ and timely detection on a community level is emphasised as the key to an effectful prevention of deaths because of undernutrition. Knowledge about high-risk sub-populations in the community makes it possible to direct these community interventions. Such focused strategies are already recommended when establishing preventive measures for child undernutrition, such as supplementary food support or cash transfers.^7,8^

This study hypothesised that children living in households inhabiting a household member who suffers from life-limiting illness are at high risk of undernutrition. Such households could represent a sub-population where preventive interventions at the community-level could be relevant. We thus compared households where a household member received palliative care from Rays of Hope Hospice Jinja (RHHJ) with non-RHHJ households. We investigated the prevalence of acute malnutrition among the children, below the age of 5 years, in these households. Furthermore, we investigated the association between child nutritional status and documented risk factors for acute malnutrition, including household dietary diversity,^9^ household food insecurity^10^ and household healthcare barriers.

## Research methods and design

### Study design

A comparative cross-sectional study was conducted, with comparison of households that receive treatment from RHHJ (RHHJ households) and households that do not receive treatment from RHHJ (non-RHHJ households).

The study assessed nutritional status of children below 5 years of age and assessed household dietary diversity, household food insecurity and household healthcare barriers.

### Study site and risk households

Rays of Hope Hospice Jinja is a non-governmental-organisation (NGO) based in Jinja district, eastern region, Uganda. RHHJ’s catchment area is the whole of Busoga region and a few neighbouring districts; approximately 10.000 km^2^. Rays of Hope Hospice Jinja provides holistic palliative care to patients and their families with severe life-limiting illnesses. The assessment of severe, life-limiting illness is based on a holistic investigation made by medical workers at RHHJ. At the time of data collection, 29% had cancer, 26% were untreated or had a treatment failure for HIV / AIDS, 33% had cancer (any stage) and HIV and 12% were suffering from other illnesses (chronic wounds, osteomyelitis, etc.). Eligible patients are identified by volunteer community workers, who collaborate closely with RHHJ.

### Inclusion and exclusion criteria

#### Inclusion in the Rays of Hope Hospice Jinja household group

Households that received palliative care from RHHJ because of severe, life-limiting illness and had at least one child under the age of 5 years who had lived with them for at least 8 weeks were included.

#### Inclusion in the non-Rays of Hope Hospice Jinja household group

Households that did not receive care from RHHJ and had at least one child under the age of 5 years who had lived with them for at least 8 weeks were included. The non-RHHJ households were matched on neighbourhood.

#### Exclusion criteria

Children who according to caregivers suffered from either HIV / AIDS or cancer were excluded (If the parents were HIV negative, it was assumed that the child was HIV negative but if one parent was HIV positive, medical records with a test confirming HIV negative status for the child(ren) was required for inclusion). Individuals who declined participation were also excluded.

### Selection of research participants

#### Rays of Hope Hospice Jinja households

The eligible households were contacted by phone. Patients who confirmed to have children under five and were willing to participate in the study were visited for data collection.

#### Non-Rays of Hope Hospice Jinja -households

The households were selected by asking the neighbouring household with children under five if they were interested in participation in the study. The neighbourhood families were identified either by the RHHJ driver or by the patient in a RHHJ household.

### Methods of data collection

Children living in RHHJ households and non-RHHJ households, respectively, had their overall nutritional status evaluated. We determined whether children suffered from acute malnutrition and/or stunting in accordance with the WHO cut-offs.^11^ The following anthropometric measurements were used: Weight (kg), height (cm), age (years and months), Mid-Upper Arm Circumference (MUAC) (mm) and presence of bilateral pedal pitting oedema that could not be explained by a known medical condition. Acute malnutrition is divided into moderate acute malnutrition (MAM) and severe acute malnutrition (SAM). In children between 6 and 59 months, MAM is defined as MUAC ≥ 115 mm and < 125 mm or weight for height (WHZ) between –3 and –2 standard deviations (s.d.) of the population mean.^12^ Severe acute malnutrition is defined as MUAC < 115 mm or WHZ < –3 or bilateral pedal pitting oedema.^13^ Stunting is defined as length-for-age or height-for-age < –2 s.d (9).

Each measure was repeated three times and on this basis an average was calculated. Equipment used for measurements were: SECA 874 (flat scale), SECA 217 (stadiometer), SECA 417 (infantometer) and MUAC tape. The SECA 874 flat scale enabled parent–child-weight-measuring whenever the child was too young to stand or had difficulty cooperating for independent weighing.

The socioeconomic status and information on household dietary and food security status were obtained with the questionnaires Household Dietary Diversity Score (HDDS),^9^ and Household Food Insecurity Access Scale (HFIAS).^10^ Furthermore, the healthcare barrier assessment was developed based on the Uganda Vulnerability Index Assessment (2014).^14^ All questions were answered by a household member who had a clear idea of the food consumption of the household. When possible, we interviewed the primary responsible person for the household diet.

### Training of data collectors

Two research assistants helped with data collection. They were trained in all methods used before the initiation of data collection and supported by a brush-up session in the middle of the collection process.

#### Refinement of questionnaire

The research team refined the questionnaire on the arrival of the PI in Uganda. Refinement included refinement of extent and phrasing. The questionnaire was tested on local subjects to make sure questions were understood correctly and to make sure the time frame for executing the questionnaire interview was realistic. After final refinement, the questionnaire was translated to Luganda by a professional translator to ensure streamlined phrasing of questions throughout the data collection.

#### Data handling and confidentiality

Data were obtained by the PI and four RHHJ staff members and managed in REDCap. The PI was responsible for collection of all data and for ensuring standardised data collection. Access to REDCap was provided via Aarhus University, Denmark. All staff members from RHHJ have signed a confidentiality agreement.

#### Coronavirus disease 2019 risk management plan

All those involved, including study participants and research staff were given personal protective gear to protect them from coronavirus disease 2019 (COVID-19). Commitments to work within the standard operating procedures were made, which included wearing masks, sanitising regularly, maintaining a social distance and keeping the meetings with small numbers.

#### Study population size

Based on observations made by the RHHJ staff we estimate the prevalence of acute malnutrition (either SAM or MAM) to be 15% in children in households where a household member receives palliative care. Based on Uganda Demographic and Health Survey 2016, 3.6% of children < 5 years in the Busoga region suffer from acute malnutrition (–2 s.d.). With 90% power and a 95% confidence interval, we will need to include a minimum of 152 households from each cohort to show a possible association. Although there possibly is more than one child under five in a household, we still need a minimum of 152 households to avoid confounding.

#### Compensation of research participants

Rays of Hope Hospice Jinja households who participated in this study were given a food basket worth 20 000 Ugandan shillings and the non-RHHJ households were given a food basket worth 10 000 Ugandan shillings.

For households where we discovered that a child or children had malnutrition in the form of either SAM or MAM, the families were given nutritive porridge in addition to the food basket, and health education about a balanced diet and early medical intervention in case the child was ill.

### Statistics

Data were analysed using R for Windows version 4.1.0. To create Table 1 the package ‘tableone’ was used.^15^ To test for differences in means between groups, linear mixed effects modelling was used to allow for the clustering design on the household-level; the ‘lme4’ package was used.^16^ To calculate prevalence estimates and z-score statistics on anthropometric data, the ‘anthro’ package was used, which is based on methodology recommended by WHO and UNICEF.^17^ Chi-square tests were used to observe differences in characteristics in the two groups. Two sample *t*-test was used to test for statistically significant differences in mean for total HDDS and total health care barrier score.

**TABLE 1:** Background characteristics of study participants.

Household characteristic	Non-RHHJ households	RHHJ households	*p*
**Children**	*N* = 213	*N* = 226	
Female sex (*n*(%))	112 (52.6)	108 (47.8)	0.36
Age, months (mean(s.d.))	29.3 (16.1)	32.6 (16.0)	0.04
**Households**	*N* = 145	*N* = 145	-
Children < five per household (mean (s.d.))	1.54 (0.72)	1.65 (0.83)	0.21
Persons per household (mean (s.d.))	6.32 (3.24)	8.15 (4.79)	< 0.001
**Main household income earner (*n*(%))**			< 0.001
Father	110 (75.9)	61 (42.4)	-
Mother	26 (17.9)	37 (25.7)	-
Relatives	4 (2.8)	29 (20.1)	-
Grandparent or elderly	4 (2.8)	12 (8.3)	-
Child	1 (0.7)	5 (3.5)	-
**Main source of income (*n*(%))**			0.09
Formal business/commercial farming/formal employment	15 (10.3)	13 (9.0)	-
Petty business	18 (12.4)	17 (11.7)	-
Casual labourer/informal employment/Peasantry/Hiring out labour on other farms/gardens	111 (76.6)	105 (72.4)	-
Remittances	1 (0.7)	5 (3.4)	-
None	0 (0.0)	5 (3.4)	-
**Access to land (*n*(%))**			0.50
Owns and able to access land	104 (71.7)	107 (73.8)	-
Owns but not able to access land	1 (0.7)	4 (2.8)	-
Does not own but able to access land	23 (15.9)	20 (13.8)	-
Does not own and no access to land	17 (11.7)	14 (9.7)	-

Note: RHHJ Households: Households receiving care from Rays of Hope Hospice Jinja. The table is created in *R* with the table one function package; For characteristics with two groups a *t*-test was performed to calculate the *p*-value. For characteristics with > 2 subgroups (i.e. main source of income) a chi^2^-test was performed to calculate the *p*-value.

RHHJ, Rays of Hope Hospice Jinja; s.d., standard deviation.

### Ethical considerations

Ethical clearance to conduct this study was obtained from the Hospice Africa Uganda Research and Ethics Committee (HAUREC) (No. HAU-2021-5). We obtained informed consent for both anthropometric measurements and for collection of information on nutrition, healthcare barriers and socio-economic parameters. Respondents were able to ask questions in their own language (Luganda) before consenting. We obtained signatures or fingerprints for informed consents; if the parent or guardian was illiterate a witness’s signature was obtained. All research subjects or authorised surrogate decision-makers were given written information about the research in the research subjects’ own and non-technical language. The study abided by ‘International Ethical Guidelines for Epidemiological Studies’ prepared by the Council for International Organizations of Medical Sciences (CIOMS) in collaboration with the WHO (2008).

## Results

**FIGURE 1:**
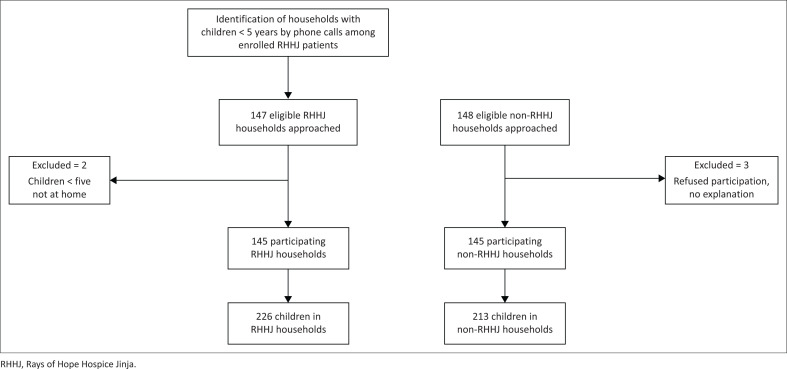
Diagram for research participants.

Between April and July 2021, a total of 290 households and 439 children aged between 0 and 59 months were included in the study. There were 226 children from RHHJ households and 213 children from non-RHHJ households (Figure 1).

The mean age of children below 5 years of age was significantly higher in the RHHJ households than the non-RHHJ households. The number of household members was significantly higher in the RHHJ households than the non-RHHJ households. The two cohorts differed in *main household income earner*, as the father was the main income earner in 75.9% (110/145) of the non-RHHJ households whereas 42.4% (61/144) of the RHHJ households had the father as main income earner (Table 1).

### Prevalence of malnutrition

Table 2 shows the prevalence of malnutrition-indicators. The prevalence of acute malnutrition was 4.5% in the total study population. Of all 20 children suffering from acute malnutrition 12 children were from RHHJ households and eight were from non-RHHJ households. The prevalence of stunting was 30.8% in both the RHHJ households and non-RHHJ households.

**TABLE 2:** The prevalence of malnutrition indicators.

Variable	RHHJ households prevalence (CI95)	Non-RHHJ households prevalence (CI95)	Total prevalence (CI95)
**Any acute malnutrition**	5.3% (12/226)	3.8% (8/213)	4.5% (20/439)
**Moderate acute malnutrition**			
MUAC < 12.5 cm ≥ 11.5 cm	2.7% (6/226)	1.9% (4/213)	2.3% (10/439)
Weight-for-height z-score	1.8% (4/226)	0.9% (2/211)	1.4% (6/437)
**Severe acute malnutrition**			
MUAC < 11.5 cm	0.4% (1/226)	0.5% (1/226)	0.5% (2/439)
Weight-for-height *z*-score and/or bilateral oedema	1.8% (0.7; 4.7) (4/226)	1.9% (0.7; 5.0) (4/211)	1.8% (0.9; 3.6) (8/437)
Bilateral pitting oedema	1.3% (3/226)	1.9% (4/213)	1.6% (7/439)
Wasting	2.2% (5/226)	0.9% (2/211)	1.6% (7/437)
Stunting	30.8% (25.0; 37.2) (68/221)	30.8% (24.8; 37.4) (64/208)	30.8% (26.6; 35.3) (132/429)

Note: RHHJ Households: Households receiving care from Rays of Hope Hospice Jinja. MUAC: Mid–Upper Arm Circumference. Acute malnutrition: Weight–for–height *z*–score < –2 or bilateral pitting oedema or MUAC < 125 mm. Moderate acute malnutrition: Weight–for–height *z*–score < –2 and ≥ –3 or MUAC < 125 mm and ≥ 115 mm. Severe acute malnutrition: Weight–for–height *z*–score < –3 or bilateral pitting oedema or MUAC < 115 mm. Wasting: Weight–for–height *z*–score < –2. Stunting: length–for–age/height–for–age *z*–score < –2.

RHHJ, Rays of Hope Hospice Jinja; MUAC, Mid-Upper Arm Circumference; Cl, Confidence interval.

**FIGURE 2:**
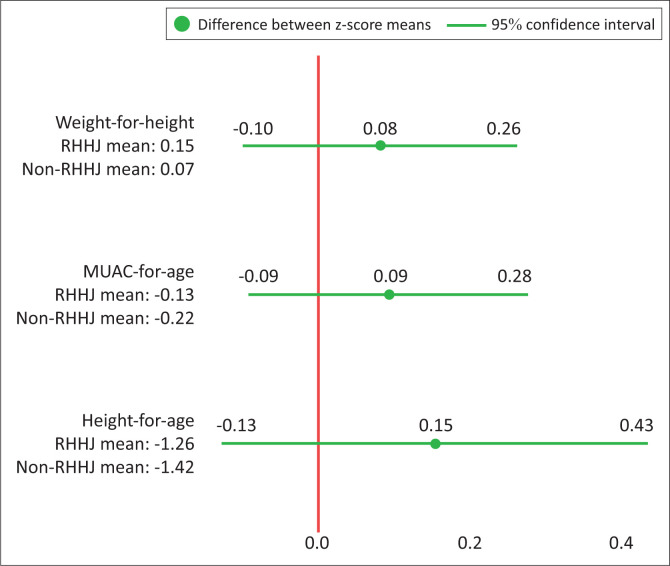
Test of difference between nutritional status in household groups.

The difference in mean *z*-scores between the two cohorts was not statistically significant for any of the nutrition indicators: Weight-for-height, height-for-age and MUAC-for-age (Figure 2). All indicators showed a trend towards higher *z*-scores in the RHHJ households than in the non-RHHJ households, but as it isn’t statistically significant, such trends should not be concluded upon.

### Assessment of household food diversity, household food security and household healthcare barriers as risk factors for undernutrition

**FIGURE 3:**
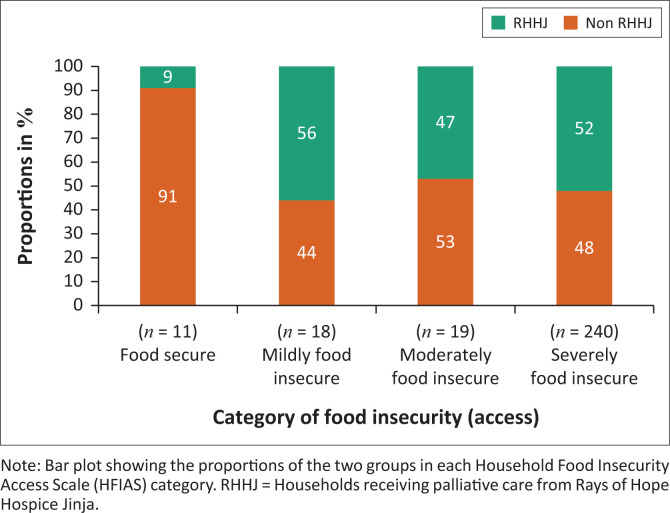
Household food insecurity.

The household food insecurity assessed by HFIAS depended on the household group, demonstrated by a Chi-square test (χ^2^) (*p* = 0.048); thus, the RHHJ households were associated with food insecurity (Figure 3).

No association was found between dietary diversity, assessed by HDDS or healthcare barriers and any of the continuous malnutrition indicators (weight-for-height, height-for-age, MUAC-for-age) when tested with random effects linear regression (Table 3).

**TABLE 3:** Correlation between risk exposures and nutritional indicators.

Variable	Slope coefficient	95% CI	*p*
**Household food diversity, HDDS**			
Weight-for-height *z*-score	−0.006	(−0.04; 0.04)	0.98
Height-for-age *z*-score	0.002	(−0.05; 0.06)	0.95
MUAC-for-age *z*-score	−0.033	(−0.07; 0.00)	0.08
**Household health care barriers**			
Weight-for-height *z*-score	−0.006	(−0.05; 0.04)	0.79
Height-for-age *z*-score	−0.023	(−0.09; 0.04)	0.47
MUAC-for-age *z*-score	−0.020	(−0.06; 0.02)	0.35

MUAC, Mid-Upper Arm Circumference; HDDS, Household Dietary Diversity Score; Cl, Confidence interval.

Table 3 showed the correlation between suggested risk exposures (HDDS and healthcare barriers) and anthropometric measures for diagnosing malnutrition (weight-for-height, height-for-age, MUAC-for-age). Tested by random effects linear regression on data for the total population, with the null hypothesis reflected as the slope coefficient = 0. *T*-test was performed to assess *p*-value for slope coefficient = 0.

A *p* < 0.05 is considered statistically significant. No statistically significant correlation between HDDS/healthcare barriers and any of the nutrition indicators was found.

The HFIAS is not normal distributed, and therefore not included in this analysis although it is considered a risk exposure for malnutrition.

## Discussion

We investigated whether there is an increased risk of child undernutrition in Ugandan rural households where a household member has a life-limiting illness and concluded that undernutrition is not more prevalent in these households.

To our knowledge this is the first study to investigate nutritional status of children below the age of 5 years in households inhabiting a patient with a life-limiting illness such as cancer and/or insufficiently treated HIV / AIDS.

### Lower overall prevalence of acute malnutrition than expected

According to the Uganda Demographic and Health Survey 2016, 3.6% of children < 5 years in Busoga region suffer from acute malnutrition. We found a lower prevalence in our study. There are several possible explanations as to why we found lower overall prevalence of wasting (weight-for-height < –2) in both RHHJ and non-RHHJ households than expected.^18^

One crucial explanation may be the season, as data collection was performed during the wet season and the dry season had been short in 2021 leading to successful harvests during data collection. A study from Tanzania has demonstrated seasonal variation reflected as higher admission to the hospital with acute malnutrition and lower mean weight-for-height *z*-score (0.15[0.10; 0.20]) during the dry season.^19^ Similarly, a study in rural Ethiopia demonstrated high incidence of SAM and low recovery rates in the post-harvest season.^20^

In our study, research participants had good access to nutritious food during data collection. Moreover, a successful harvest secures income generation for many of the households, as most of the study participants had ‘casual labourer/informal employment/Peasantry/Hiring out labour on other farms/gardens’ as the main source of income (Table 1). In summary, the season dictates short-term access to nutritious food and income generation in the rural areas, which is where RHHJ primarily provides health services. If the data collection had been performed during the dry season, we may have found higher rates of acute malnutrition.

Seasonal variation in food accessibility is supported by the relatively high prevalence of stunting in both groups (30.8%), which may indicate that children have insufficient nutrient intake part of the year. A similar high prevalence of stunting in rural areas has been shown in a study investigating undernutrition of adolescent girls in Ethiopia with a prevalence of 24.2% in rural areas compared to 16% in urban areas,^21^ possibly explained by the seasonal variation in access to food.

### No increased risk of acute malnutrition in Rays of Hope Hospice Jinja households

The hypothesis of increased risk of acute malnutrition in RHHJ households, was in part developed because of a suspected higher degree of poverty in households receiving help from RHHJ compared to the non-RHHJ households. This is also reflected in the HFIAS assessment (Figure 3), which indicates a higher degree of food insecurity in RHHJ households than non-RHHJ households. In spite of this, there is not an increased risk of acute malnutrition; on the contrary we see a tendency towards higher *z*-scores in all nutritional indicators in the RHHJ households (see Figure 2), although this trend is not statistically significant. This may be explained by a protective effect provided by RHHJ who ensure better access to healthcare for all household members, despite their primary task, which is to provide palliative care for the household member with a life-limiting illness. There are several mechanisms for how better access to healthcare for the children can be conveyed. As RHHJ ensures frequent home-based patient visits, the staff of RHHJ reports that families often ask for advice regarding health concerns for family members other than the patient. In other words, RHHJ’s presence in the patient homes gives access to medications (given out for free, if it is part of RHHJ’s basis medication), early diagnosis of diseases, help navigating the health system, help with referrals in the health system, financial help for transport and counselling on general child health. A study in rural Malawi demonstrated how a 21-day training programme related to complementary feeding, water, sanitation and hygiene significantly improved *z*-scores for wasting and undernutrition.^22^ Rays of Hope Hospice Jinja’s home-based visits may possibly constitute a similar effect on child nutrition because of their presence and counselling on child nutrition and health, although it should be emphasised that the intervention in Malawi was a specific intervention on nutrition. Furthermore, the children in the household benefit if the household is assessed to be eligible to receive food support from RHHJ (approximately 20% of the households enrolled into the RHHJ programme are assessed to be eligible for food support).

### Household level risk factors of child undernutrition

No association between child nutritional status and following household level explanatory variables were found: Household Dietary Diversity, Household Food Insecurity and household health care barriers.

The higher risk of food insecurity in the RHHJ households (Figure 3) may indicate that the RHHJ households are more vulnerable in terms of food security. So even though the prevalence of undernutrition is low in the RHHJ households, the nutritional status would presumably quickly deteriorate without the food support and increased access to health care provided by RHHJ.

## Limitations

Firstly, the cross-sectional study design does not allow for conclusions about causality. Secondly, our selection of a comparison group, the non-RHHJ households, may introduce some bias, as in some cases we had help from a RHHJ household member to identify a non-RHHJ household, and explain the purpose of our study. We considered this selection method necessary in some cases, as in some areas we experienced heavy scepticism and a fear of exploitation if participating in the study. Whenever this selection method was used, we countered bias by thoroughly asking about all neighbours with children under the age of five, so the data collection team randomly could select a comparison non-RHHJ household, and in addition we stressed the purpose of research rather than charity. However, in most areas several neighbours lived in feasible proximity, which enabled the driver participating in the data collection to identify a non-RHHJ household by approaching a random non-RHHJ household and invite for study participation.

Thirdly, the use of four different data collectors introduced the risk of bias with regard to streamlining of data handling. This was counted for with training sessions for the included data collectors, and in addition the PI was part of data collection for 282/290 households, which ensured streamlining of data handling. Fourthly, the questionnaires for the estimation of household healthcare barriers have not been validated. Fifthly, the COVID-19 pandemic lockdowns may have affected the household economies, as lockdowns hindered some people from working.

## Recommended further research

The potential protective effect of RHHJ healthcare services on child nutrition should further be investigated to identify key elements responsible. Such elements could be upscaled in the RHHJ programme and others similar to RHHJ. More importantly, other social – or health aid programmes can gain from this knowledge and include elements in their routines and support prevention of acute malnutrition among children at risk.

## Conclusion

The overall prevalence of wasting (low WHZ) among children under 5 years was low in the Busoga region (overall) when compared to a demographic survey from 2016 (1.4% compared to 3.6% below WHZ –2s.d.).^18^

Almost one third of all included children suffered from stunting, which is in alignment with the 2016 survey.^18^

The mean *z*-scores for nutritional indicators did not significantly differ between households receiving palliative care from RHHJ and non-RHHJ households, respectively. Although non-statistically significant, a trend towards higher *z*-scores for anthropometric nutritional indicators was observed in households receiving palliative care from RHHJ.

Household food insecurity was statistically significantly higher in households receiving palliative care from RHHJ than in non-RHHJ households.

Further studies are recommended to investigate a potential protective effect on child nutritional status when receiving home-based aid.
